# Prevalence of TB-related symptoms and self-reported disability among adult TB survivors

**DOI:** 10.5588/ijtldopen.24.0141

**Published:** 2024-12-01

**Authors:** A.K. McDonald, D. Nakkonde, P. Kaggwa, S. Zalwango, A. Joseph, E. Buregyeya, J.N. Sekandi

**Affiliations:** ^1^ College of Public Health, Global Health Institute, University of Georgia, Athens, GA, USA;; ^2^ Department of Epidemiology and Biostatistics, College of Public Health, University of Georgia, Athens, GA, USA;; ^3^ Makerere University, School of Public Health, College of Health Sciences, Kampala, Uganda;; ^4^ Directorate of Public Health Service and Environment, Kampala Capital City Authority, Kampala, Uganda.

**Keywords:** tuberculosis, post-TB treatment, TB-associated disability, post-TB morbidity, TB survivors, Uganda, low-income settings

## Abstract

**BACKGROUND:**

Growing evidence suggests that post-TB-related morbidity occurs often among TB survivors, but there is limited epidemiological data on the burden of symptoms and disability after successful completion of treatment. We evaluated the prevalence of TB-related symptoms, self-reported disability, and factors associated with disability among adult TB survivors who recently completed treatment in Uganda.

**METHODS:**

Between January 2022 and October 2023, we conducted a study of adults who completed treatment for drug-susceptible TB in Kampala, Uganda. We collected data on demographics, TB-related symptoms, HIV status, and disability measured using the World Health Organization Disability Assessment Schedule (WHODAS 2.0).

**RESULTS:**

Of the 200 participants, the median age was 33.0 years (IQR 26–44.5); 52.5% were male, and 23% were HIV-infected. The prevalence of TB symptoms was 58%, and self-reported disability was 83.5%. Factors significantly associated with disability were having completed treatment within the last 6–8 months and experiencing TB symptoms (aOR 2.87, *P* = 0.04; and aOR 2.51, *P* = 0.03, respectively), after adjusting for age, sex and HIV status.

**CONCLUSIONS:**

TB-related symptoms and self-reporting of any disability were highly prevalent in the study population. There is a need for further longitudinal evaluation and considerations to expand the continuum of care and support to improve the quality of life for TB survivors post-TB treatment.

## RÉSUMÉ

**CONTEXTE:**

De plus en plus de preuves suggèrent que la morbidité post-tuberculeuse est fréquente chez les survivants de la TB, mais il existe peu de données épidémiologiques sur le fardeau des symptômes et de l'invalidité après la réussite du traitement. Nous avons évalué la prévalence des symptômes liés à la TB, de l'invalidité autodéclarée et des facteurs associés à l'invalidité chez les survivants adultes de la TB qui ont récemment terminé leur traitement en Ouganda.

**MÉTHODES:**

Entre janvier 2022 et octobre 2023, nous avons mené une étude sur des adultes qui ont terminé un traitement contre la TB sensible aux médicaments à Kampala, en Ouganda. Nous avons recueilli des données sur les données démographiques, les symptômes liés à la TB, le statut VIH et l'incapacité mesurées à l'aide du calendrier d'évaluation de l'invalidité de l'Organisation mondiale de la Santé (WHODAS 2.0, pour l’anglais « WHO Disability Assessment Schedule, version 2.0 »).

**RÉSULTATS:**

Sur les 200 participants, l'âge médian était de 33,0 ans (IQR 26–44,5) ; 52,5% étaient des hommes et 23% étaient infectés par le VIH. La prévalence des symptômes de la TB était de 58% et celle de l'invalidité autodéclarée était de 83,5%. Les facteurs significativement associés à l'incapacité étaient le fait d'avoir terminé le traitement au cours des 6 à 8 derniers mois et de présenter des symptômes de la TB (aOR 2,87 ; *P* = 0,04 ; et aOR 2,51 ; *P* = 0,03, respectivement), après ajustement pour tenir compte de l'âge, du sexe et de la séropositivité.

**CONCLUSIONS:**

Les symptômes liés à la TB et l'auto-déclaration d'un handicap étaient très répandus dans la population étudiée. Il est nécessaire de procéder à une évaluation longitudinale plus poussée et d'envisager d'élargir le continuum de soins et de soutien afin d'améliorer la qualité de vie des survivants de la TB après le traitement de la TB.

TB is a major global health issue, causing millions of deaths annually. In 2021, there were 10.6 million incident TB cases and 1.6 million TB-related deaths;^1^ 30 countries with high TB burdens had 87% of new cases, mainly in low- to middle-income countries.^1^ Uganda, a low-income country with a high TB burden, had 200 cases/100,000 people and a death rate of 35/100,000 in 2019.

Effective TB treatment exists, yet its impact extends beyond initial complications.^2,3^ Studies reveal post-treatment issues like impaired lung function, abnormal radiological findings, and reduced quality of life.^4-7^ TB-related disability can stem from the disease itself or treatment side effects, often linked to the affected site (e.g., pulmonary TB affects lung function, and spinal TB leads to paraparesis).^8,9^ TB medications may also cause visual/hearing impairments^10^ and even mental disorders.^11^

Although TB-related disability is recognised, its prevalence and nature are poorly understood.^2,3,6^ The accurate quantification of post-TB treatment disabilities is crucial for policy-making and service provision globally.^2,12^ Understanding post-treatment disability could also prevent future disabilities in patients with TB.^2^ Recent evidence suggests that the burden of TB is underestimated because the current estimates of disability-adjusted life-years (DALYs) and years of healthy life lost due to disability (YLDs) do not fully account for post-TB disability.^13^ Moreover, studies indicate that quality of life is negatively affected post-TB treatment due to complications associated with either the treatment of TB disease or the illness itself.^2,12,13^

Additionally, TB-affected households sustain financial hardships both during and post-treatment.^14^ A study by Meghji and others posits that post-TB morbidity is associated with limited financial recovery due to conditions acquired and experienced post-treatment.^15^ Therefore, there is a need for continued assessment and support post-TB treatment,^2–4^ especially in low-income settings. The support should target improvements in the quality of life for TB survivors as well as the economic viability of their households. Our study aimed to determine the prevalence of TB-related symptoms, self-reported disability and the factors associated with disability post-treatment in a cohort of patients in Kampala, Uganda.

## METHODS

### Ethical considerations

The study obtained approval from the institutional review boards at the University of Georgia, Athens, GA, USA; and Makerere University School of Public Health, Kampala, Uganda. Participants provided written informed consent for the post-treatment interview, given in English or Luganda based on their preference. Participants received ∼US$10 for travel and compensation for their time.

### Study setting and population

Our study was conducted in Kampala, Uganda, among participants who were previously enrolled in the ‘DOT Selfie’ randomised controlled trial of video directly observed therapy (VDOT) and usual care DOT (UCDOT) from July 2019 to December 2021.^16^ Specific data for this analysis was collected from January 2022 to October 2023. Collaboration between researchers at Makerere University School of Public Health (Kampala, Uganda), the University of Georgia (Athens, GA, USA), and the public health clinics supervised by the Kampala Capital City Authority (Kampala, Uganda) and the National TB Programme staff was crucial for this study’s success. Kampala, the capital of Uganda, has the highest incidence of TB.^17^ Diagnosis, treatment, and care of TB are government-funded, and services are provided free to all patients in Uganda.

### Study design, eligibility and sampling

A cross-sectional study was conducted among adult participants aged 18 years and older who had completed the ‘DOT Selfie’ (VDOT) trial and others who completed routine TB treatment for drug-susceptible TB at least 6 months before data collection. Participants who had been treated for drug-resistant TB were not eligible for the study or those who did not provide consent to participate. A convenience sample of eligible TB survivors was contacted by phone for the post-treatment study. Due to limited resources, this study was set up as a pragmatic exploratory pilot to generate hypotheses to inform well-designed and powered future studies as the next steps. In the end, 200 participants were located and agreed to participate in the post-TB treatment interviews.

### Participant recruitment, data collection and study procedures

**Figure 1.**
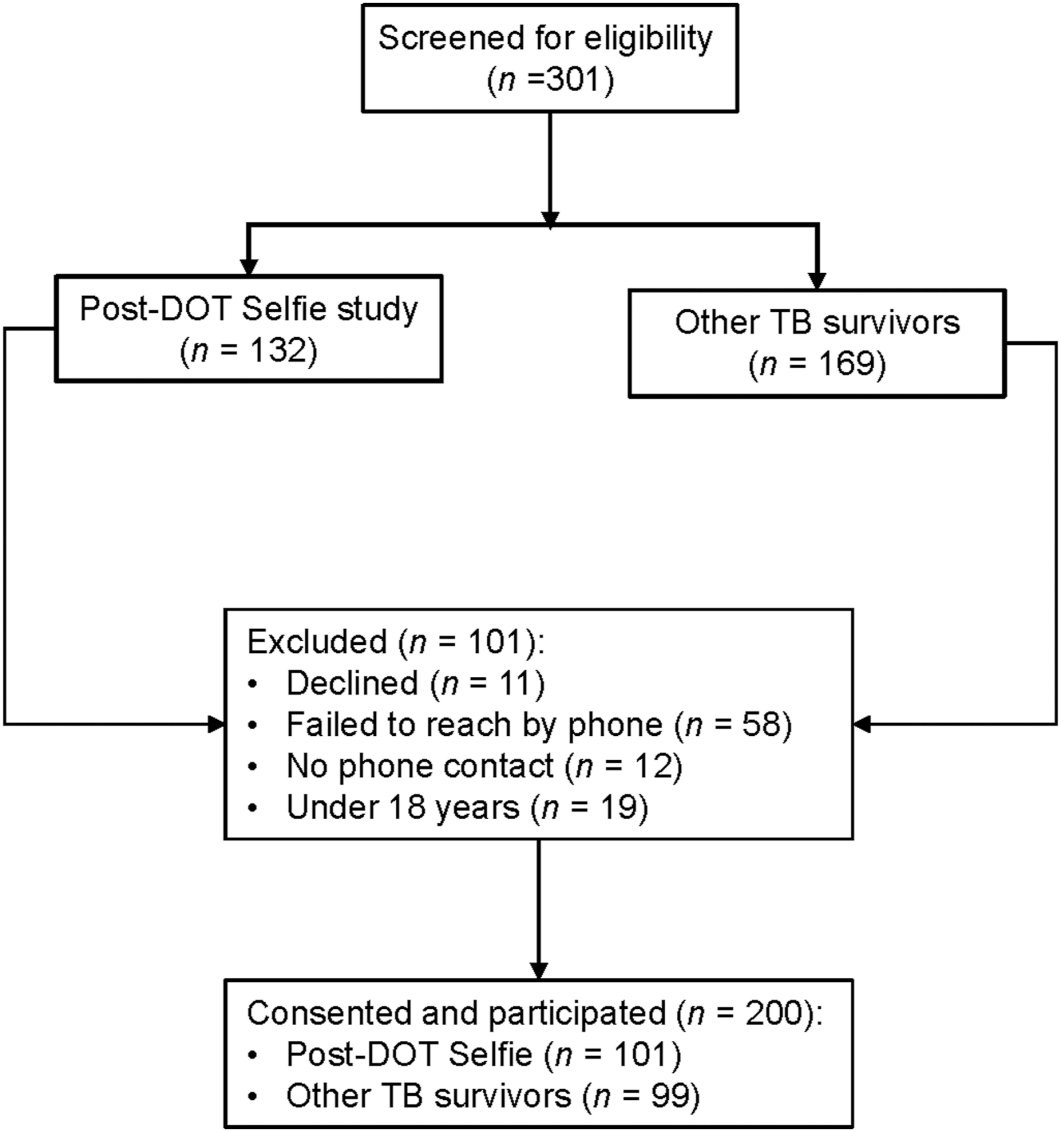
Flow diagram of enrolled post-TB participants. DOT = directly observed therapy.

Participants were screened for eligibility, and informed consent was obtained through a two-step process (Figure 1). First, participants who had completed treatment either as part of the ‘DOT Selfie’ study or routine treatment were traced by phone calls. They received a brief overview of the post-TB study to gauge their interest in returning to the clinic. Second, participants were requested to voluntarily return to the TB clinic, where they were informed of the post-TB study, and then the written informed consent was fully administered to those willing to participate. All except four surveys were completed at the TB clinic in Lubaga Hospital, Kampala, Uganda. The four participants (two men travelling from Kampala City and two outside of the country) were interviewed via cell phone due to their distance from the clinic. This method was deemed appropriate given that there were no required in-person study procedures, and we had also obtained approval to use the phone interview option during the original study as part of the required COVID-19 risk mitigation plan.

Trained interviewers used structured questionnaires to collect the data on sociodemographics and current clinical status, including persistent and new TB-related symptoms such as cough, chest pain, appetite loss, weight loss, fevers, and night sweats. Participants also answered specific questions about self-reported disability using a 12-item scale based on the WHO Disability Schedule v2.0 (WHODAS 2.0).

### World Health Organization Disability Assessment Schedule (WHODAS)

The WHODAS 2.0, developed by the WHO, assesses health and disability^18^ across six domains of functioning: cognition, mobility, self-care, getting along, life activities, and social participation. The full-length questionnaire contains 36 questions; the shorter version is composed of 12 questions with two questions per domain, employing a five-point Likert scale to assess the level of difficulty in the completion of the specified tasks.^18^ According to Chiang et al.,^19^ the WHODAS was created based on The International Classification of Functioning, Disability and Health framework (ICF). The reliability and applicability of the WHODAS 2.0 were tested globally,^18,20,21^ with translations into 47 languages, including African ones such as Xhosa, Xitsonga, Lusoga (from Uganda), Yoruba, and Zulu.^22^ We aimed to gain an initial understanding of the prevalence and the magnitude of the self-reported disability.

### Data analysis

Data were analysed using SAS v9.4 M8 (SAS Institute, Cary, NC, USA), presenting baseline characteristics using descriptive statistics like frequencies, mean, standard deviation (SD), median, interquartile range (IQR), and percentages. The outcome variable disability was a dichotomous composite disability variable (Yes, No). To generate the variable, the category ‘yes’ included all persons reporting any disability in any of the six domains measured in the WHODAS 2.0 scale. The category ‘No’ included all persons who self-reported the absence of problems in all six domains. We took a simplistic approach to the analysis just because the study was not powered for more detailed analyses. We also analysed disability as a categorical variable with (mild, moderate, and severe disability) using multinomial logistic regression, but some cells were very small, yielding less interpretable results. In the end, the most meaningful analysis was the one from the dichotomous variable, which was useful for interpretation and comparison with existing studies.

The primary exposure variable, TB-related symptoms, was also characterised as a dichotomous variable (Yes, No); Yes, one or more symptoms were reported, and no, no symptoms were reported. Simple and multivariable logistic regression assessed disability’s relationship with TB symptoms, sex, age, employment, HIV, previous TB diagnosis, location, and health status. All observations were included. We reported crude (cOR) and adjusted odd ratios (aOR) with 95% CI, considering predictors with *P* < 0.05 as significant.

## RESULTS

### Characteristics of participants

We screened 301 participants, of whom 132 were from the ‘DOT Selfie’ (VDOT) study and 169 from the routine TB care clinics, as shown in the study flow diagram (Figure 1). A total of 200 participants were enrolled with a median age of 33.0 years (IQR 26.0–44.5), ranging from 19 to 70 years. Most were male (52.5%), and 23% had HIV. Most (84%) completed TB treatment at least 9 months before the interview and were employed (78%). Baseline characteristics of study participants are shown in Table 1.

**Table 1.** Baseline characteristics of participants in Kampala, Uganda (*n* = 200).

Characteristics	*n* (%)
Total enrolled	200 (100)
Sex	
Female	95 (47.5)
Male	105 (52.5)
Age, years, median [IQR]	33.0 [26.0–44.5]
Age groups, years	
18–34	110 (55.0)
35–44	40 (20.0)
45–70	50 (25.0)
Time since treatment completion, months	
6–8 months	32 (16.0)
≥9 months	168 (84.0)
Self-rated health status	
Good-very good	140 (70.0)
Bad-moderate	60 (30.0)
HIV status	
Positive	46 (23.0)
Negative	154 (77.0)
TB-related symptoms	
Yes	116 (58.0)
No	84 (42.0)
Place of residence	
Kampala	133 (66.5)
Outside Kampala	67 (33.5)
Highest level of education attained	
None/primary 1–7	63 (31.5)
Senior and secondary 1–6	93 (46.5)
Certificate/diploma/tertiary/university	44 (22.0)
Religion/denomination	
Catholic	60 (30.0)
Protestant	56 (28.0)
Muslim	45 (22.5)
Pentecostal	30 (15.0)
Seventh Day Adventist or other	9 (4.5)
Employment status	
Yes	156 (78.0)
No	44 (22.0)
Work for pay	
Yes	166 (83.0)
No	34 (17.0)
Household monthly income, USD,^†^ mean ± SD	289.2 ± 666.3
Personal monthly income, USD,^†^ mean ± SD	129.8 ± 229.8

*Multiple responses allowed.

† 1USD = UGX3762.90.

IQR = interquartile range; USD = US dollar; UGX = Ugandan shilling.

### Prevalence of TB-related symptoms

A total of 140 (70%) participants self-reported a health status ranging from good to very good, and 58% reported experiencing one or more symptoms post-TB treatment. The most prevalent symptom was cough (51%), and the least reported was night sweats and blood in sputum at 4% and 2%, respectively. Only 16.5% of the participants reported more than one symptom.

### Prevalence of self-reported disability

**Figure 2.**
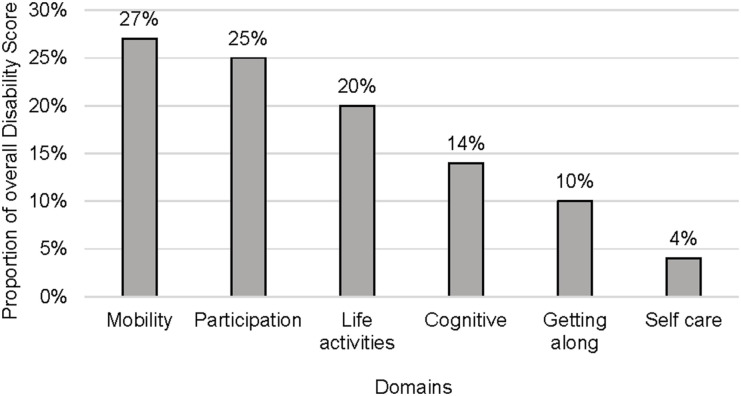
Prevalence of domain-specific disability.

**Figure 3.**
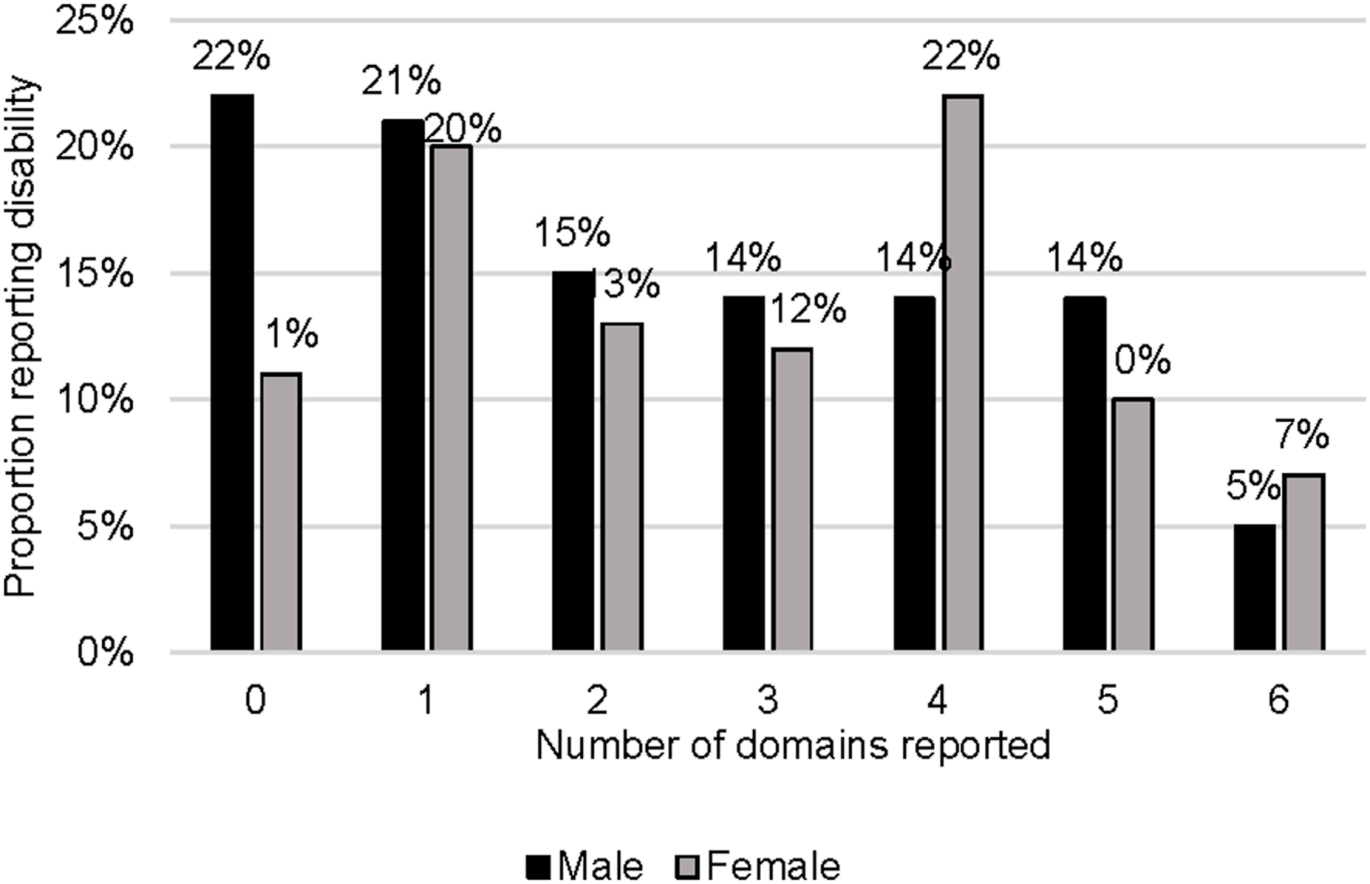
Prevalence of disability by the number of domains chosen stratified by sex.

As shown in Table 2, the majority (83.5%) reported disability in one or more domains, with a median score of 16 (IQR 16–21). Disability in the mobility domain was most common (58.5%), contributing significantly to the cumulative score (27%). Prevalence in other domains was cognition (42%), self-care (9.5%), getting along (30%), life activities (50%), and participation (65.5%), as shown in Figure 2. Mobility and participation contributed most to the cumulative score (53%). Males and females had similar domain frequencies and median scores (males: 15, IQR 13–20; females: 16, IQR 13–22), as displayed in Table 2. Supplementary Table S1 in the Supplementary Data outlines disability levels (none, mild, moderate, high). Most participants (38%) fell into the low level (total score 13–16/60) among those reporting disabilities (Figures 2 and 3).

**Table 2.** Distribution of median disability scores based on factors associated with disability.

Characteristics	*n*	Disability Score Median [IQR]
Total	200	16 [13–21]
Sex		
Male	105	15 [13–20]
Female	95	16 [13–22]
Age, years		
18–34	110	16 [13–21]
35–44	40	17 [13–21]
45–70	50	16 [12–19]
TB-related symptoms		
Yes	156	15 [13–18]
No	84	17 [14–23]
HIV status		
Positive	46	15 [12–21]
Negative	154	16 [13–21]
Employment status		
Employed	156	16 [13–20]
Unemployed	44	20.5 [15–25]
Self-reported health status		
Bad-moderate	60	20 [15–24]
Good-very good	140	15 [13–18]
Treatment completion, months		
6–8	32	20 [15–24]
≥9	168	15 [13–18]

IQR = interquartile range.

### Factors associated with self-reported disability

Females had double the likelihood of reporting disability (cOR 2.02, 95% CI 0.92–4.44), while HIV-negative individuals were also twice as likely (cOR 2.23, 95% CI 1.00–4.99). Participants with TB symptoms were three times more likely to report disability (cOR 2.54, 95% CI 1.18–5.46), along with participants completing treatment 6–8 months prior (cOR 3.31, 95% CI 1.21–9.00). After adjusting for age, TB symptoms, treatment completion, sex, and HIV status, TB symptoms and treatment completion remained significant factors, with aORs of 2.39 (95% CI 1.06–5.42) and 2.95 (95% CI 1.04–8.38), respectively (Table 3).

**Table 3.** Logistic regression analysis of factors associated with post-TB disability.

		Disability		cOR (95%CI)	*P*-value	aOR (95%CI)	*P*-value
Predictor variables		Yes	No				
Sex	Male	83	22	1.00	—	1.00	—
	Female	84	11	2.02 (0.92–4.44)	0.08	2.31 (0.98–5.45)	0.06
Age, years	18–34	95	15	2.23 (0.96–5.13)	0.06	1.47 (0.58–3.73)	0.41
	35–44	35	5	2.46 (0.79–7.62)	0.12	2.18 (0.66–7.17)	0.20
	45–70	37	13	1	—	—	—
TB-related symptoms	Yes	104	13	2.48 (1.15–5.32)	0.02	2.39 (1.06–5.42)	0.04
	No	63	20	1.00	—	1.00	—
HIV status	Positive	34	12	1.00	—	1.00	—
	Negative	133	21	2.23 (1.00–4.99)	0.04	2.37 (0.96–5.84)	0.06
Employment status	Employed	129	27	1.00	—	—	—
	Unemployed	38	6	1.33 (0.51–3.45)	0.56	—	—
Self-reported health status	Bad–moderate	55	5	2.75 (1.01–7.51)	0.05	—	—
	Good–very good	112	28	1.00	—	—	—
Treatment completion, months	6–8	62	5	3.31 (1.21–9.00)	0.02	2.95 (1.04–8.38)	0.04
	≥9	105	28	1.00	—	1.00	—
TB history	New case	133	24	1.48 (0.63–3.45)	0.38	—	—
	Retreatment	34	9	1.00		—	—
Location	Kampala	112	21	1.16 (0.53–2.54)	0.70	—	—
	Outside of Kampala	55	12	1.00	—	—	—

cOR = crude odds ratio; CI = confidence interval; aOR = adjusted OR.

Other covariates were related to disability but lacked statistical significance. For example, individuals reporting fair to moderate health status had higher odds of reporting disability (cOR 2.75, 95% CI 1.01–7.1), as shown in Table 3. Health status was not factored into adjusted calculations due to a strong correlation with symptoms.

## DISCUSSION

In this study, we determined the prevalence of self-reported TB-related symptoms and disability among TB survivors who had completed treatment for drug-susceptible TB at least 6 months before enrollment in Kampala, Uganda. We found that both TB-related symptoms and self-reported disability were highly prevalent in the study populations. To our knowledge, this is one of the first studies to evaluate the prevalence of TB-related symptoms, disability, and the associated factors in Uganda. Our findings generally confirm some of the results on disability from a recent multi-country study to determine disability, comorbidities and risk determinants at the end of treatment in Kenya, Uganda, Zambia and Zimbabwe.^23^ There were some differences in the findings of the two studies, mainly explained by the measurements used. Our findings suggest that despite the ‘successful’ treatment of TB disease, patients still need support to improve their quality of life as part of the continuum of care in the post-TB period.

Our study found that more than half (58%) of participants reported one or more symptoms related to TB, predominantly chronic cough post-treatment. Additionally, reporting one or more TB-related symptoms was significantly associated with reporting self-reported disability. A recent study by Meghji and her colleagues also found that participants who reported TB symptoms were more likely to report disability one year post-treatment.^7^ The most prevalent symptom in our study was cough. Similarly, studies conducted in South Tamil India, Malawi, Nigeria, Ethiopia, Tanzania, and the Democratic Republic of Congo show that respiratory symptoms are widespread among drug-susceptible TB patients during and post-treatment.^2,7,11,24–26^ The prevalence of respiratory symptoms may be due to TB healing by fibrosis, which results in residual respiratory impairments. The respiratory impairments affect the quality of life of TB survivors, hamper their reintegration into society, and their economic viability post-treatment.^7,15^

In our study, the vast majority (83.5%) of TB survivors reported disability in one or more of the six domains of functioning. Similar findings were found in a study done in Peru, where at least 68% of participants had some disability post-treatment.^10^ However, the Peru study did not use the WHODAS 2.0 scale to measure disability; instead, it used the Washington Group short-set questions on functioning. In addition, most participants predominantly reported disability in two domains: social participation (65.5%) and mobility (58.5%). These two domains contributed to more than half of the cumulative disability score for the study population.

For the high disability scores in the social participation domain, a possible explanation could be the persistence of self-stigma associated with TB, which lingers on even after survivors have completed treatment. Studies show that there is still a high prevalence of perceived, self-, and experienced stigma towards TB patients.^2,27,28^ This stigma can affect a patient’s motivation to engage in group/community activities during and post-treatment.^2,24,29^ Stigma in all its forms can affect not only an individual’s mental health but also their economic viability due to being unable to find and keep work.^27,30^ Therefore, there may be a need for continued education among TB survivors and communities about stigma after treatment to garner social support for reintegration into society.

The relationship between sex and disability was not statistically significant in our study. However, we found that women were more likely to report disability than men and had higher overall median disability scores. In a survey completed in Malawi, women experienced most of the disability burden post-TB treatment.^13^ Previous studies have shown that women are more likely than men to have adverse events or worse outcomes.^13,31,32^

Studies conducted in sub-Saharan Africa and Pakistan have suggested that the increased risk of negative outcomes among females can be attributed to the undertreatment and underdiagnosing of women due to structural, social, and cultural factors within countries.^33,34^ Therefore, there is a need for gender-specific interventions that account for the increased risks seen among women in high-burden settings.

Time since TB treatment completion was associated with self-reported disability, with 6–8 months being at a greater risk compared to more than 8 or more months duration since completion. Our findings are concordant with the study by Nightingale and colleagues, which showed that YLDs due to TB decrease as time since successful TB treatment increased.^6^ Therefore, it is important that TB-related disability is addressed soon after treatment completion to minimise its impact on the quality of life of TB survivors.

HIV status was a significant independent factor associated with self-reported disability. However, after adjusting for confounding by TB symptoms, the association was attenuated by time since treatment completion, sex, and age. Our results point to a possible clinical impact of HIV status on self-reported disability. Similarly, a study conducted in urban Malawi among a prospective cohort of drug-susceptible pulmonary TB patients found that being HIV-positive was protective against TB-related disability.^13^ This finding may be counterintuitive given the known impact of HIV on a person’s health.^35^ Our results may be due to a small sample size, with only 23% being HIV-positive.

Additionally, persons living with HIV might likely have had a longer duration of poor quality of life such that they could have a higher threshold for reporting disability. Another closely related explanation could be that due to HIV, people could have been suffering very similar symptoms or disability before the TB diagnosis and didn’t see the need to report. This scenario could have led to an underreporting of symptoms and disability.

### Strengths and limitations

We acknowledge that our study has some strengths and limitations. One key strength is that we used the WHODAS 2.0 to assess disability, which has been widely validated even in Africa. To date, most published studies have used limited scales that only assess disability that impacts physical well-being post-TB treatment. Our study was limited in using the short WHODAS 2.0 version of 12 -questions; however, a larger study using the 36-item schedule could shed further light on the burden of post-TB disability and allow for a more robust analysis.

Second, we used a cross-sectional design that does not allow for the assessment of a causal relationship between key variables and disability.

It is conceivable that the self-reported disability existed before diagnosis and persisted throughout the TB treatment but was only documented following treatment completion. Therefore, we recommend future well-designed cohort studies that assess and compare disability across the entire spectrum. Additionally, we used a convenience sample, limiting our results’ generalisability. Finally, we did not assess the effect of the severity of TB disease on disability, which would have further clarified the high-risk groups for disability among TB survivors. The Uganda National TB programme services at public clinics use a variety of diagnostics depending on availability and clinicians’ decisions, making it difficult to make meaningful comparisons on severity as a variable. However, our findings provide a reasonable foundation for well-designed future observational studies.

## CONCLUSION

TB-related symptoms and self-reporting of any disability were highly prevalent in the study population. There is a need for further longitudinal evaluation and considerations to expand the continuum of care and support to improve the quality of life for TB survivors post-TB treatment.

## Supplementary Material


