# Supplementary material for: Prevalence of TB-related symptoms and self-reported disability among adult TB survivors
Source: IJTLD Open. 2024 Dec 1;1(12):540–6. doi: 10.5588/ijtldopen.24.0141 (PMC11636498; doi:10.5588/ijtldopen.24.0141)
Supplement: Supplementary file 1 [file ijtldopen24-0141_supplementarydata1.docx]

# Prevalence of TB-related symptoms and self-reported disability among adult TB survivors

**Supplemental Table S1. Distribution of disability severity by factors associated with disability post TB treatment**

| Characteristics | Severity of Disability | | | | |
| --- | --- | --- | --- | --- | --- |
|  | **None** | **Low** | **Moderate** | **High** | **p-value** |
| Total |  |  |  |  |  |
| Sex  Male  Female | 22 (11.0)  11 (5.0) | 39 (19.5)  37 (18.5) | 22 (11.0)  22 (11.0) | 22 (11.0)  14 (25.0) | 0.33 |
| Age  18-34  >34 | 15 (7.5)  18 (9.0) | 45 (22.5)  31 (15.5) | 23 (11.5)  21 (10.5) | 27 (13.5)  20 (10.0) | 0.58 |
| TB-related symptoms  Yes  No | 20 (10.0)  13 (6.5) | 34 (17.0)  42 (21.0) | 18 (9.0)  26 (13.0) | 11 (5.5)  36 (18.0) | <0.01 |
| HIV status  Positive  Negative | 12 (6.0)  21 (10.5) | 17 (8.5)  58 (29.5) | 8 (4)  36 (18.0) | 9 (4.5)  38 (19.0) | 0.26 |
| Employment status  Employed  Unemployed | 27 (13.5)  6 (3.0) | 65 (32.5)  11 (5.5) | 32 (16)  12 (6.0) | 32 (16.0)  15 (7.5) | 0.10 |
| Self-rated Health Status  Bad-Moderate  Good-Very Good | 5 (2.5)  28 (14.0) | 15 (7.5)  61 (30.5) | 14 (7.0)  50 (15.0) | 26 (13.0)  21 (10.5) | <0.01 |
| Treatment Completion  Less than 9 months  9 months or more | 5 (2.5)  28 (14.0) | 23 (11.5)  53 (26.5) | 19 (9.5)  25 (12.5) | 20 (10.0)  27 (13.5) | 0.03 |

***Fisher’s exact p-value**
